# Dissemination and Comparison of Genetic Determinants of *mcr*-Mediated Colistin Resistance in Enterobacteriaceae *via* Retailed Raw Meat Products

**DOI:** 10.3389/fmicb.2019.02824

**Published:** 2019-12-12

**Authors:** Tereza Gelbíčová, Alžběta Baráková, Martina Florianová, Ivana Jamborová, Markéta Zelendová, Lucie Pospíšilová, Ivana Koláčková, Renáta Karpíšková

**Affiliations:** ^1^Department of Bacteriology, Veterinary Research Institute, Brno, Czechia; ^2^Faculty of Science, Masaryk University, Brno, Czechia; ^3^Faculty of Veterinary Medicine, University of Veterinary and Pharmaceutical Sciences Brno, Brno, Czechia; ^4^Central European Institute of Technology, University of Veterinary and Pharmaceutical Sciences Brno, Brno, Czechia

**Keywords:** colistin, *mcr* gene, resistance, Enterobacteriaceae, meat, retail

## Abstract

The global food chain may significantly promote the dissemination of bacteria resistant to antibiotics around the world. This study was aimed at determining the prevalence and genetic characteristics of Enterobacteriaceae with *mcr*-mediated colistin (CT) resistance in retail meat of different origins. Bacteria of the Enterobacteriaceae family carrying the *mcr-1* gene were detected in 21% (18/86) of the examined samples, especially in turkey meat and liver originating from EU and non-EU countries (19%) and in rabbit meat imported from China (2%). The examined samples of the meat and liver of chicken and other poultry and of pork and beef were negative for the presence of bacteria carrying the *mcr-1* to *mcr-5* genes. A huge number of isolates belonging to *Escherchia coli* (*n* = 54), *K*lebsiella *pneumoniae* (*n* = 6), and *Citrobacter braakii* (n = 1) carrying the *mcr-1* gene were obtained. Despite the high heterogeneity of the tested isolates, the *mcr-1* gene was localized on only three types of plasmids (IncX4, IncHI2, and IncI2). The most frequent type of plasmid was IncX4, which carried the *mcr-1* gene in 77% of *E. coli* and *K. pneumoniae* isolates from turkey meat and liver samples from the Czechia, Germany, Poland, and Brazil. Our findings indicate highly probable interspecies transfer of IncX4 and IncI2 plasmids within one meat sample. The co-resistance of plasmid-mediated CT resistance encoded by the *mcr-1* and ESBL genes was detected in 18% of the isolates. Another noteworthy finding was the *fosA3* gene coding for fosfomycin resistance in a multidrug-resistant isolate of *E. coli* from rabbit meat imported from China. The observed high level of Enterobacteriaceae with plasmids carrying the *mcr-1* gene in retail meat reflects the need for Europe-wide monitoring of *mcr*-mediated CT resistance throughout the whole food chain.

## Introduction

The dissemination of plasmid-mediated colistin (CT) resistance poses a substantial health concern to both human and veterinary medicine. Plasmid-mediated CT resistance encoded by the *mcr-1* gene was first reported in China in Enterobacteriaceae from various sources (Liu et al., 2016). Since then, there has been a worldwide spread of gram-negative bacteria with the *mcr-1* gene, especially *Escherichia coli*, in animals including food animals, food, the environment, and humans (Skov and Monnet, 2016; Al-Tawfiq et al., 2017). Another mobile CT resistance gene, *mcr-2*, has been described in *E. coli* isolated from pigs and cattle in Belgium (Xavier et al., 2016). However, its occurrence in other countries has been reported only rarely (Zhang et al., 2018). In 2017, the following four genes were described: (i) *mcr-3* in *E. coli* from pigs in China (Yin et al., 2017); (ii) *mcr-4* in *Salmonella* and *E. coli* isolated from pigs in Italy, Spain, and Belgium (Carattoli et al., 2017); (iii) *mcr-5* in *Salmonella* Paratyphi B from poultry in Germany (Borowiak et al., 2017); and (iv) *mcr-6* (originally termed *mcr-2.2* variant) in *Moraxella pluranimalium* from pigs at slaughter in the United Kingdom (AbuOun et al., 2018). The next year, *mcr-7* was detected in *Klebsiella pneumoniae* from chickens and *mcr-8* in *K. pneumoniae* from pigs, chickens, and humans in China (Wang X. et al., 2018; Yang et al., 2018). In 2019, the *mcr-9* gene was described in CT-susceptible *Salmonella* Typhimurium isolated from a patient in the United States in 2010. However, expression of *mcr-9* in *E. coli* resulted in phenotypic resistance to CT (Carroll et al., 2019). The most recently found *mcr* gene is *mcr-10* in *Enterobacter roggenkampii* (GenBank, accession number MN179494.1).

Extensive use of CT in veterinary medicine for infection control or prophylaxis contributed to a significant increase in the prevalence of CT-resistant bacteria (Kempf et al., 2016). In human medicine, CT is used as an antibiotic of last resort in patients infected with multidrug-resistant gram-negative bacteria (Al-Tawfiq et al., 2017). *E. coli* isolates with both chromosomal and plasmid-mediated CT resistance in patients without a history of CT treatment have been detected in China. The fact that more than half of them were farmers suggested the possibility of resistant bacteria transmission to humans in association with agricultural use of antimicrobials (Luo et al., 2017). The level of total sales of antimicrobials to treat farm animals in the Czechia is low compared to in Spain, Italy, Germany, and France (ESVAC, 2018). However, the sales and consumption of antibiotics by farm animals may not correspond to the occurrence of resistant bacteria in foods of animal origin in a given country. The global animal and food market can also contribute to the spread of bacteria resistant to the main classes of antibiotics. The spread of *mcr-1* is associated with various types of plasmids, including IncI2, IncX4, IncF, IncHI1, IncHI2, IncP, and IncY. The most prevalent plasmid types are IncI2 and IncX4 (Wang R. et al., 2018). The high similarity of plasmids carrying *mcr-1* in *E. coli* isolated from patients and from poultry meat in Switzerland indicates a role of certain types of “epidemic” plasmids (IncI2 and IncX4) in *mcr-1* gene dissemination along the food chain and in humans (Zurfluh et al., 2017).

In a number of European countries, the prevalence of gram-negative bacteria with *mcr* genes was found to be higher in poultry, especially turkeys, compared to pigs and cattle (Irrgang et al., 2016; Perrin-Guyomard et al., 2016; Alba et al., 2018). In 11 European countries, the prevalence of the *mcr-1* gene between 2002 and 2014 was shown to be 1.2% in CT-resistant *E. coli* from chickens and 0.7% in isolates from pigs. By contrast, *mcr-1* was not detected in any isolates from cattle. These *E. coli* isolates with the *mcr-1* gene were detected mainly in Spain and Germany and also in Netherlands and France. In Germany, all *E. coli* isolates with the *mcr-1* gene were detected in poultry, whereas in Spain, they originated from pigs (El Garch et al., 2018). On the other hand, in Germany, Roschanski et al. (2017) described the occurrence of *E. coli* with *mcr-1* in 9.9% of pig samples from 25.9% of pig farms examined. However, direct PCR testing of genes without sample enrichment showed a high prevalence of *mcr-1* in China and the presence of *mcr-2*, *mcr-3*, *mcr-4*, and *mcr-5* in rectal/cloacal and nasal/oropharyngeal swabs from pigs and poultry (Chen et al., 2018; Zhang et al., 2018). The *mcr-1* gene was identified in up to 79.2% samples from pigs and in 31.8% of poultry samples (Zhang et al., 2018). Overall though, it is difficult to compare data on the prevalence of plasmid-mediated CT resistance in individual countries due to differences in the methodological approaches used in various studies.

Plasmid-mediated CT resistance in Enterobacteriaceae with a predominance of *mcr-1* has also been detected in retailed meat, particularly in chicken and turkey meat (Hasman et al., 2015; Irrgang et al., 2016; Kluytmans-van den Bergh et al., 2016; Monte et al., 2017). Furthermore, the occurrence of Enterobacteriaceae with *mcr-1* has been reported in pork (Liu et al., 2016) and beef (Mulvey et al., 2016). However, there is still a lack of comprehensive information concerning the occurrence of plasmid-mediated CT resistance in foodborne bacteria. Therefore, this study aimed at determining the prevalence and characteristics of bacteria in the Enterobacteriaceae family carrying *mcr* genes that had been isolated from retail meat from different animal species (poultry, pork, beef, and rabbit).

## Materials and Methods

### Examined Samples

Randomly selected meat samples were collected from those currently on offer at retail markets in the Czechia. A total of 86 meat and liver samples from various animal species were examined that originated from the Czechia (*n* = 29), Poland (*n* = 19), Hungary (*n* = 8), Germany (*n* = 6), Slovakia (*n* = 4), France (*n* = 4), Austria (*n* = 2), Spain (*n* = 1), Netherlands (*n* = 1), Belgium (*n* = 1), Great Britain (*n* = 1), Brazil (*n* = 8), and China (*n* = 2). These samples included pork (*n* = 10), beef (*n* = 19), mixed ground pork and beef (*n* = 5), turkey meat and liver (*n* = 24), the meat and liver of chicken (*n* = 15) and other poultry [goose (*n* = 2), duck (*n* = 4), quail (*n* = 1)], and rabbit meat (*n* = 6). Five sub-units of 25 g were collected from each sample to increase the detection rate. The samples were cultivated overnight under aerobic conditions in buffered peptone water at 37°C. After enrichment, 1 ml was collected from each sample, and DNA was isolated using a Blood and Tissue Kit according to the manufacturer’s instructions (Qiagen, Germany). The presence of the *mcr-1* to *mcr-5* genes was verified by PCR (Liu et al., 2016; Xavier et al., 2016; Borowiak et al., 2017; Carattoli et al., 2017; Yin et al., 2017). The artificially synthesized positive controls for *mcr-1* to *mcr-5* (GeneArt Strings synthesis, Thermo Fisher Scientific, United States) were prepared according to the original sequences (Liu et al., 2016; Xavier et al., 2016; Borowiak et al., 2017; Carattoli et al., 2017; Yin et al., 2017) and were used together with a negative control (sterilized ultrapure water instead of DNA) in PCR reactions. PCR-positive samples were subsequently inoculated on agar medium and cultured, thus allowing selective detection of CT-resistant bacteria and subsequent identification of *mcr* genes by PCR as described below.

### Detection of Colistin-Resistant Bacterial Isolates Carrying *mcr* Genes

In order to detect CT-resistant Enterobacteriaceae, *Brilliance* UTI Clarity agar (Oxoid, United Kingdom) with the addition of CT sulfate (Discovery Fine Chemicals, United Kingdom) was used at a final concentration of 3.5 μg/mL and incubated overnight at 37°C. In morphologically different colonies (1–8 per plate), the respective *mcr* gene was detected with the PCR method according to the results of PCR detection in meat samples after enrichment, as mentioned above.

### Species Identification of Bacterial Isolates

The *mcr*-positive isolates obtained from the UTI Clarity agar supplemented with CT were identified by matrix-assisted laser desorption ionization-time-of-flight mass spectrometry (MALDI-TOF MS) with the use of Biotyper software (version 3.1, Bruker Daltonics GmbH, Germany).

### Colistin Susceptibility Testing

The minimum inhibitory concentration (MIC) of CT was determined by the microdilution method recommended by EUCAST (2018). Microtitration plates (LabMediaServis, CZ) were designed to test 15 different concentrations of CT: 0.25, 0.5, 1, 2, 4, 8, 16, 32, 64, 128, 256, 512, 1024, 2048, and 4096 mg/L.

### Pulsed-Field Gel Electrophoresis

The Enterobacteriaceae isolates were subjected to DNA macrorestriction analysis followed by pulsed-field gel electrophoresis (PFGE) using restriction endonuclease *Xba*I according to the PulseNet Europe (2013) protocol for *E. coli* (2013). BioNumerics v5.1 (Applied Maths), with the settings Dice (Opt: 1.10%) (Tol 1.0–1.0%) (H > 0.0% S > 0.0%) (0.0–100.0%), was used to construct an unweighted pair group method with arithmetic mean (UPGMA) dendrogram.

### Whole-Genome Sequencing

For whole-genome sequencing (WGS), strains of different pulsotypes (one band difference) were selected from each positive meat sample. Genomic DNA was isolated using the Blood and Tissue Kit according to the manufacturer’s instructions (Qiagen, Germany). The preparation of DNA libraries and sequencing on the Illumina platform were carried out by Eurofins Genomics (Miseq 2 × 300 bp, *n* = 9), Macrogen Korea (Hiseq X Ten 2 × 300 bp, *n* = 20), and CEITEC VFU (Miseq 2 × 250 bp, *n* = 32). Assembly was performed with SPAdes 3.9 software^1^. The *E. coli* sequence type (ST) was determined by the Achtman MLST scheme^2^, whereas the Pasteur MLST scheme was used for *K. pneumoniae* strains^3^. Plasmid types and resistance genes were evaluated by PlasmidFinder (Carattoli et al., 2014) and ResFinder (Zankari et al., 2012)^4^. Screening for the presence of *mcr-1* genes on a particular plasmid type was performed by *in silico* analysis of the generated contigs.

The sequence data obtained were assembled using Velvet version 1.1.04 on Ridom SeqSphere+ (version 3.5.0; Ridom GmbH, Münster, Germany). The isolates were compared using MLST core genome (cgMLST) analyses comprising 2513 loci^5^ (for *E. coli*) and 2568 loci^6^ (for *K. pneumoniae*). Subsequently, phylogenetic trees for the given bacterial strain were constructed by UPGMA analysis. The threshold cluster identification was ≤10 alleles for *E. coli* and ≤15 alleles for *K. pneumoniae* according to the Ridom SeqSphere+ software.

### Conjugation Assay of Plasmids Carrying the *mcr-1* Gene

Conjugation assays were performed to determine the transferability of *mcr* genes into plasmid-free sodium azide-resistant *E. coli* J53 K12 recipient cells using the filter-mating method (Borowiak et al., 2019). The transconjugants (TCs) were selected on LB agar plates (LB) with sodium azide (100 mg/L) and CT (1 mg/L). In selected strains, the TCs were obtained on LB with sodium azide (100 mg/L) and one of the following antibiotics: sulfonamide (512 mg/L), tetracycline (16 mg/L), or ampicillin (16 mg/L). The antibiotics used for the selection were chosen according to the resistance profiles of the donor strains and WGS-based *in silico* analysis of *mcr-*carrying plasmids. In particular, the selection of TCs only on medium with CT was performed for isolates for which the confirmed *mcr* location was on either IncX4 or IncI2. If no TCs were obtained, the selection was repeated using LB with the above-mentioned antibiotics according to the donor’s resistance profile. For isolates carrying the gene on IncHI2, selections were performed using all four main antibiotics (Supplementary Table 1). Successful transfer of the resistance plasmid was confirmed by PCR targeting the *mcr-1* gene (Lescat et al., 2018) and by specific PCR for identification of the *E. coli* J53 K12 (Bauer et al., 2007). The size of plasmids transferred to recipient cells was estimated by S1 nuclease PFGE analysis (Jamborova et al., 2015), and plasmids were classified into incompatibility (Inc) groups by PCR-based replicon typing (PBRT; Carattoli, 2013).

## Results

### Prevalence of Enterobacteriaceae With *mcr* Genes in Raw Meat and Liver

Bacteria of the Enterobacteriaceae family carrying the *mcr-1* gene were detected in 21% (18/86) of the examined samples of originally packaged raw meat and liver of various animal species retailed in the Czechia. The *mcr-2*, *mcr-3*, *mcr-4*, and *mcr-5* genes were not detected in Enterobacteriaceae in the tested samples. The occurrence of Enterobacteriaceae with the *mcr-1* gene was found to be largely predominant in turkey meat and liver samples from the EU and non-EU countries. The presence of bacteria with the *mcr-1* gene has also been sporadically reported in rabbit meat, but only in meat imported from China. Samples of pork and beef, plus samples of the meat and liver of chicken and other poultry, were all found to be negative for the presence of bacteria carrying *mcr-1* to *mcr-5* genes (Table 1).

**TABLE 1:** Number of examined samples and samples positive for the presence of Enterobacteriaceae with *mcr-1* to *mcr-5* genes according to the type of meat/liver.

		**Detection of *mcr* genes in BPW**					
		**after enrichment**					
			
**Type of meat/liver**	**Examined samples (*n*)**	***mcr-1* (*n*)**	***mcr-2* (*n*)**	***mcr-3* (*n*)**	***mcr-4* (*n*)**	***mcr-5* (*n*)**	**Bacterial species carrying *mcr-1* (number of isolates)**
Pork	10	0	0	0	0	0	ND
Beef	19	0	0	0	0	0	ND
Mixed pork/beef	5	0	0	0	0	0	ND
Turkey meat and liver	24	16	0	0	0	0	*E. coli* (51)
							*K. pneumoniae* (6)
Chicken meat and liver	15	0	0	0	0	0	ND
Other poultry meat and liver (goose, duck, and quail)	7	0	0	0	0	0	ND
Rabbit	6	2	0	0	0	0	*E. coli* (3)
							*C. braakii* (1)

*BPW, buffered peptone water; ND, not detected.*

### Distribution of Bacterial Species With the *mcr-1* Gene and Their Origin

Three bacterial species, *E. coli* (*n* = 54), *K. pneumoniae* (*n* = 6), and *Citrobacter braakii* (*n* = 1) carrying the *mcr-1* gene were identified in the present study. *E. coli* isolates with the *mcr-1* gene were mainly detected in samples of turkey meat and liver from animals kept and slaughtered in Poland (23 isolates/6 samples positive for the *mcr-1* gene), Germany (9/3), Brazil (12/4) and also in the Czechia (7/3). *K. pneumoniae* with the *mcr-1* gene was isolated from samples of frozen turkey liver imported from Brazil (3/1) and turkey meat from the Czechia (3/1) in which co-occurrence of *mcr-1-*positive *E. coli* was also found. In addition to turkey meat, the *mcr-1* gene was found in *E. coli* (3/2) and *C. braakii* (1/1) isolated from frozen rabbit meat imported from China.

### MIC of Colistin

The phenotypic CT resistance was confirmed by the microdilution method. MICs for CT were found to be between 4 and 8 mg/L in *E. coli* isolates carrying the *mcr-1* gene. Most of the *E. coli* isolates (40/54) exhibited a CT MIC value of 4 mg/L, which was similar to *C. braakii*, whereas *K. pneumoniae* isolates usually showed higher MIC values for CT (8–64 mg/L).

### PFGE in *E. coli* and *K. pneumoniae* Isolates Carrying the *mcr-1* Gene

Pulsed-field gel electrophoresis was used to distinguish between strains of one species obtained from one sample. In the same sample of meat and liver, *E. coli* was classified in one to eight different pulsotypes, and *K. pneumoniae* belonging to two or three different pulsotypes was isolated. High diversity was demonstrated not only between isolates from the same sample but also between *E. coli* and *K. pneumoniae* isolates from meat and liver samples of different origins.

### Assignment of *E. coli* and *K. pneumoniae* Isolates With the *mcr-1* Gene to Sequence Types

**FIGURE 1:**
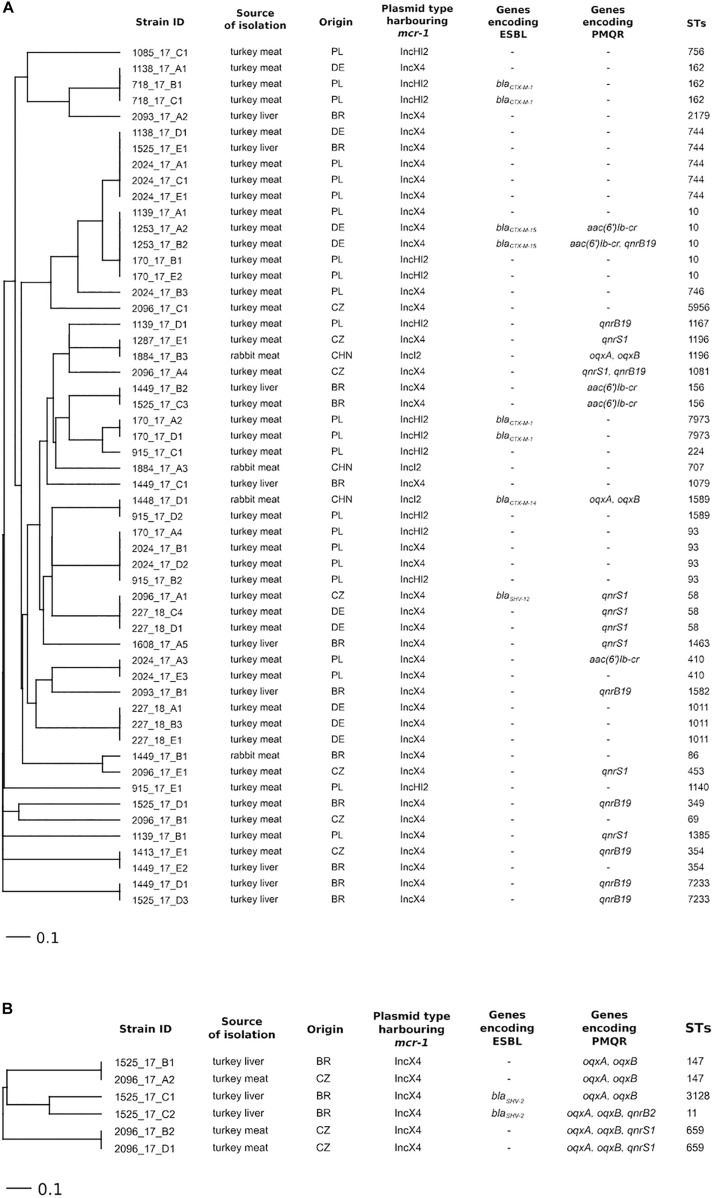
Ridom SeqSphere+ UPGMA tree based on MLST (no missing values) analyses in **(A)** *E. coli* (Achtman MLST scheme) and **(B)** *K. pneumoniae* (scheme from the Pasteur Institute) isolated from meat and liver of different origin indicating genes encoding ESBL and PMQR and plasmid types harboring the *mcr-1* gene.

*Escherichia coli* isolates (*n* = 54) were assigned to 30 different STs and *K. pneumoniae* (*n* = 6) to 4 (Figures 1A,B and Supplementary Table 1). The results did not demonstrate a correlation between the occurrence of a specific ST and the country of origin of the tested samples or the presence of a particular type of plasmid with the *mcr-1* gene. Seven STs (ST10, ST58, ST162, ST354, ST744, ST1196, and ST1589) were detected in *E. coli* from samples of raw meat and liver originating from two or more countries (Figure 1A).

### cgMLST in *E. coli* and *K. pneumoniae* Strains Carrying the *mcr-1* Gene

**FIGURE 2:**
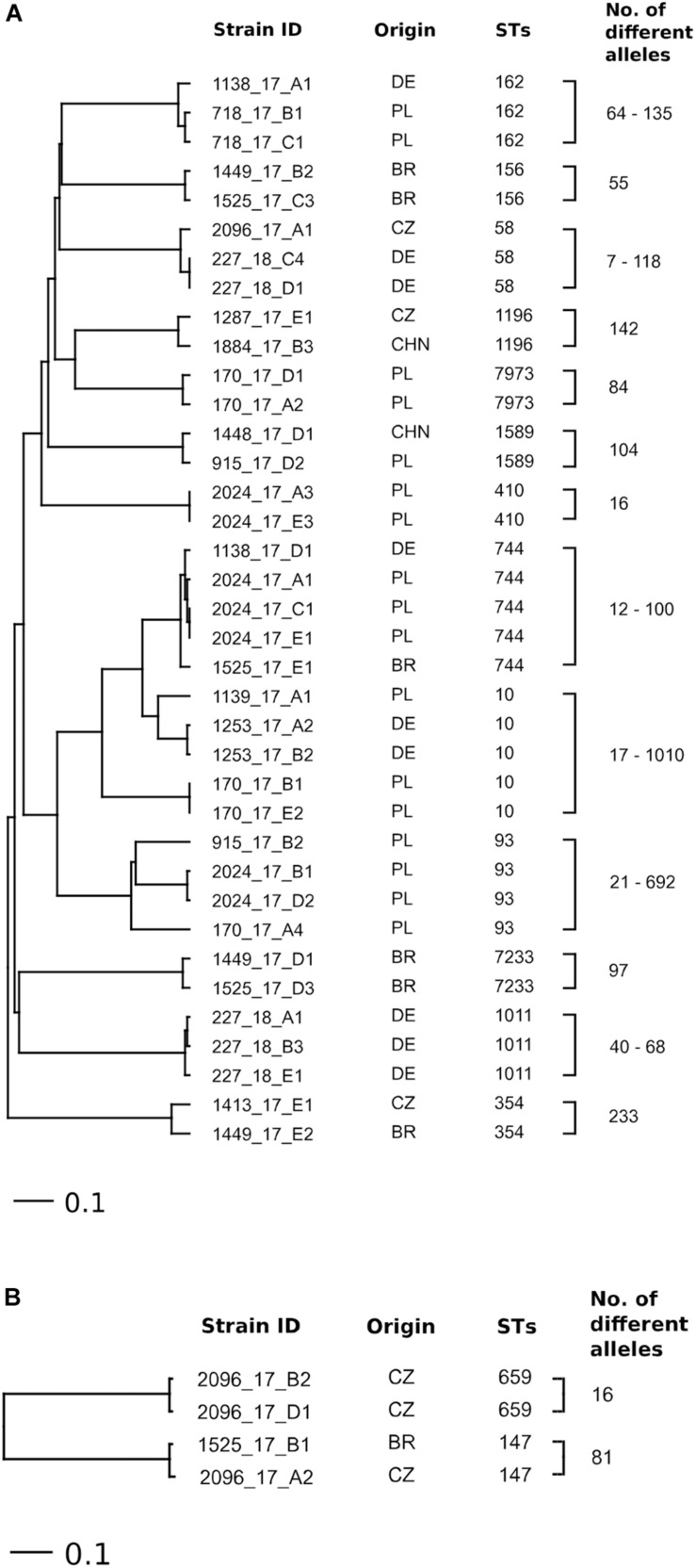
Ridom SeqSphere+ UPGMA tree based on cgMLST (missing values were considered as an own category) analyses in **(A)** *E. coli* (scheme based on 2513 loci) and **(B)** *K. pneumoniae* (scheme based on 2358 loci) strains of repeatedly detected STs isolated from meat and liver of different origin.

Based on cgMLST, high heterogeneity was confirmed among strains of *E. coli* and *K. pneumoniae* belonging to the same STs. There was no link between *E. coli* (ST10, ST58, ST162, ST354, ST744, ST1196, and ST1589) and *K. pneumoniae* (ST147) isolated from samples of meat and liver from different countries (Figures 2A,B). Only two strains of *E. coli* ST58 from one turkey meat sample from Poland were assigned to one cluster (seven allelic differences). According to PFGE analysis, these strains differed in only one band and showed a similarity of >90%. The comparison of the detected plasmids showed a difference in the presence of one additional plasmid between the strains (Supplementary Table 1). Similarly, strains of *K. pneumoniae* ST659 isolated from one turkey meat sample from the Czechia contained 16 allelic differences (close to the cluster threshold of ≤15 different alleles) and otherwise demonstrated a similarity of >90% according to PFGE analysis and differences in *bla*_SHV_ genes (Supplementary Table 1).

### Plasmids Harboring the *mcr-1* Gene in Enterobacteriaceae Isolated From Raw Meat and Liver

Based on the analysis of whole-genome sequencing data, three types of plasmids (IncX4, IncHI2, and IncI2) carrying the *mcr-1* gene were detected in the tested isolates of *E. coli*, *K. pneumoniae*, and *C. braakii*. In all isolates of *K. pneumoniae* (6/6) and the majority of *E. coli* isolates (38/51) from turkey meat and liver, the occurrence of *mcr-1* on the IncX4 plasmid type prevailed (Figures 1A,B). Samples with isolates carrying *mcr-1* on the IncX4 plasmid originated from the Czechia (seven isolates of *E. coli* and three isolates of *K. pneumoniae* from three samples), Poland (10 *E. coli* isolates from two samples), Germany (nine *E. coli* isolates from three samples), and Brazil (12 *E. coli* isolates and 3 *K. pneumoniae* isolates from four samples). *E. coli* isolates (*n* = 13) carrying the *mcr-1* gene on an IncHI2-type plasmid were detected in five out of six positive samples of turkey meat from Poland. One sample of turkey meat imported from Poland contained *E. coli* isolates (*n* = 2) with the *mcr-1* gene located on an IncHI2 plasmid and also an *E. coli* isolate with *mcr-1* on an IncX4 plasmid. The IncI2-type plasmid carrying the *mcr-1* gene was detected in only three *E. coli* isolates and one *C. braakii* isolate from two rabbit meat samples imported from China.

### Conjugative Transfer of Plasmids Carrying the *mcr-1* Gene

Conjugative transfer was revealed in all three plasmid types (IncX4, IncHI2, and IncI2) in this study. However, conjugative transfer was not confirmed in all tested isolates under the set experimental conditions. Conjugative transfer of plasmids carrying the *mcr-1* gene into recipient cells of *E. coli* was successful in 73% (27/37) of selected isolates. TCs carrying IncX4 plasmids were obtained on LB with CT (12/17). All TCs with IncI2 plasmids were selected on medium with CT (4/4). On the other hand, most TCs with IncHI2 plasmids were selected on medium with sulfonamides (7/13), ampicillin (6/17), or tetracycline (5/17); selection on LB with CT was successful only in case of one TC. S1 PFGE analysis showed that the *mcr-1* gene was carried by IncX4 of size 35–45 kb (17/37), IncHI2 of size 250–260 kb (13/37), or IncI2 of size 55–60 kb (4/37) conjugative plasmids (Supplementary Table 1). The results showed a highly probable interspecies transfer of IncX4 plasmid between *E. coli* and *K. pneumoniae* within one sample and of IncI2 between *E. coli* and *C. braakii* in another meat sample (Supplementary Table 1).

### Occurrence of Other Resistance Genes in Enterobacteriaceae Isolates Carrying the *mcr-1* Gene

Regarding the distribution of antimicrobial resistance genes, most of the *E. coli*, *K. pneumoniae*, and *C. braakii* isolates with *mcr-1* carried additional genes encoding resistance to macrolides (92%), beta-lactams (88%), tetracycline (85%), and aminoglycosides (79%) (Supplementary Table 1). Importantly, some bacterial isolates with plasmid-mediated CT resistance also carried genes coding for ESBL and PMQR. In four *E. coli* isolates and two *K. pneumoniae* isolates, the simultaneous presence of ESBL- and PMQR-encoding genes was detected (Figures 1A,B). None of the isolates carried genes encoding carbapenem resistance. Genes for ESBL production were detected in 15% (8/54) of *E. coli* isolates and in two of six *K. pneumoniae* isolates with the *mcr-1* gene. CTX-M-type ESBL was detected in *E. coli* isolates with *mcr-1* from turkey meat from Poland and Germany and rabbit meat from China. On the other hand, *E. coli* isolates from turkey meat from the Czechia carried the *bla*_SHV–__12_ gene. *K. pneumoniae* (*n* = 2) isolated from a sample of turkey liver from Brazil carried the *bla*_SHV–__2_ gene. All six obtained *K. pneumoniae* isolates with *mcr-1* and 39% (21/54) of *E. coli* isolates with *mcr-1* carried the PMQR-encoding genes. The *qnrS* and *qnrB* genes predominated in *E. coli* isolates from turkey meat and liver originating from different countries, whereas all *K. pneumoniae* isolates carried the *oqxA* and *oqxB* genes. Moreover, the *qnrS* gene was detected in two *K. pneumoniae* isolates from the Czechia and the *qnrB* gene in one isolate from Brazil. Only one *C. braakii* isolate obtained from rabbit meat from China carried both the ESBL and PMQR genes besides *mcr-1*.

## Discussion

Studies conducted on different continents report the detection of bacteria with plasmid-mediated CT resistance, but the true prevalence remains unknown. The current prevalence of bacteria with *mcr* genes in food animals and in food originating from the Czechia is unknown, because systematic and proportional studies of this type have not yet been performed. In the Czechia, the first detection of *E. coli* harboring the *mcr-1* gene was reported in 2017 in turkey meat from Poland (Karpíšková et al., 2017). A follow-up pilot study of turkey meat originating from the EU and purchased in the Czechia’s market network between 2017 and 2018 identified *mcr-1*-positive Enterobacteriaceae in 70.6% of samples (Gelbíčová et al., 2019). In the present study, 67% of turkey meat and liver samples contained *mcr-1*-positive bacteria. These samples originated from Poland, Germany, and Brazil, and also from the Czechia. Of the total *mcr-1*-positive isolates obtained from turkey meat and liver, 51 were of *E. coli* and 6 of *K. pneumoniae*. Hence, our data showed overall that turkey meat and liver produced in Poland is a major source of bacteria with plasmid-mediated CT resistance, with the highest prevalence given to *E. coli* with *mcr-1*.

These findings are consistent with a German study where the prevalence of *E. coli* with the *mcr-1* gene was higher in turkey (8.4%) than in chicken meat (4.3%) (Irrgang et al., 2016). Furthermore, this is in accordance with the higher observed prevalence of *mcr-1-*positive bacteria in turkeys from Poland, Germany, France, and Italy in comparison with broilers (Irrgang et al., 2016; Perrin-Guyomard et al., 2016; Alba et al., 2018; Zaja̧c et al., 2019).

Even though no Enterobacteriaceae species carrying *mcr* genes were found in any sample of chicken meat originating either from the EU countries (Czechia, Slovakia, Poland, Great Britain, and France) or non-EU countries (Brazil), other studies have described the occurrence of *E. coli* with *mcr-1* in retailed chicken meat. In a Swiss study, the *mcr-1* gene was detected in 25.8% of retail poultry meat (chicken and turkey) from Germany (28 samples) and Italy (5 samples). The *mcr-1* gene has not been found in any of a set of chicken and turkey meat samples from Switzerland, Denmark, Austria, and Hungary (Zurfluh et al., 2016). In Brazil, the presence of *E. coli* with *mcr-1* was detected in 19.5% of the tested retailed chicken meat and liver samples (Monte et al., 2017).

The investigated samples of retailed pork and beef from the Czechia and from other European countries have been shown to be safe regarding plasmid-mediated CT resistance. This is in spite of the fact that the presence of bacteria with plasmid-mediated CT resistance has been reported in pigs from European (El Garch et al., 2018) and from Asian (Chen et al., 2018; Zhang et al., 2018) countries, though their occurrence in pork has been described only rarely, e.g., in China (Liu et al., 2016). Similarly, the occurrence of *mcr*-positive bacteria in beef has been described only occasionally, e.g., in ground beef in Canada (Mulvey et al., 2016). The prevalence of plasmid-mediated CT resistance in bacteria isolated from cattle in Europe (Xavier et al., 2016; Hernández et al., 2017; Haenni et al., 2018) and in Asia (Kawanishi et al., 2016) is lower in comparison to poultry and pigs. CT is recommended for the treatment of gastrointestinal infections caused by non-invasive *E. coli*, mainly in pigs, chickens, turkey, calves, and sheep. However, extensive studies combining antibiotic consumption and resistance are limited because either CT resistance is not tested at all or the tests are not fully reliable (EMA/AMEG, 2016).

The dissemination of *mcr* genes in gram-negative bacteria isolated from food-producing animals and from food in Asian countries (Kawanishi et al., 2016; Liu et al., 2017; Wu et al., 2018) was confirmed by the detection of *E. coli* and *C. braakii* with the *mcr-1* gene in two samples of rabbit meat imported from China. The examined rabbit meat samples from Hungary and the Czechia did not contain bacteria with *mcr* genes. Currently, there is a paucity of studies screening for the presence of bacteria with plasmid-mediated CT resistance in rabbits and rabbit meat. In Italy, *mcr-1* was detected in 15.6% (50/320) of *E. coli* isolated from rabbits originating from 25 out of 32 investigated farms (Agnoletti et al., 2018). In Portugal, the *mcr-1* gene was detected in three *E. coli* isolates from rabbit meat (Freitas-Silva et al., 2018).

The high diversity of the *mcr-1-*positive *E. coli* and *K. pneumoniae* isolates detected in the present study corresponds to the results for *mcr-*positive Enterobacteriaceae isolated from different sources (food, animals, and humans) in other studies (Zurfluh et al., 2016; Luo et al., 2017; Roschanski et al., 2017; Zurfluh et al., 2017; El Garch et al., 2018). However, some studies have described an increased prevalence of *E. coli* with *mcr-1* belonging to ST10 obtained from food animals, food, and humans (Zurfluh et al., 2016; El Garch et al., 2018; Shen et al., 2018; Wu et al., 2018). Similarly, *E. coli* isolates found here belonging to ST10 (*n* = 5) and ST744 (*n* = 5) were most often detected. The results of cgMLST analysis provided for a more detailed differentiation compared to MLST, revealing significant differences between isolates belonging to an identical ST-type originating from one sample/country (Figures 2A,B).

Regardless of the high heterogeneity of the tested isolates, the *mcr-1* gene was localized on only three types of plasmids (IncX4, IncHI2, and IncI2), which have previously been described in association with the occurrence of *mcr-1* (Wang R. et al., 2018). The most common type of plasmid was IncX4, which carried the *mcr-1* gene in 77% (44/57) of *E. coli* and *K. pneumoniae* isolates from turkey meat and liver samples (*n* = 16) from the Czechia, Germany, Poland, and Brazil. IncX4 plasmids carrying *mcr-1* have also been described in *E. coli* isolated from meat and humans in China (Liu et al., 2017; Luo et al., 2017; Shen et al., 2018), chicken meat from Brazil (Monte et al., 2017), chicken and turkey meat from the EU (Hasman et al., 2015; Alba et al., 2018), and pigs in Germany (Roschanski et al., 2017). The conjugation assay showed that the majority of IncX4 plasmids carrying *mcr-1* were transferable to recipient *E. coli* cells (14/17). The different origin of these isolates suggesting that these plasmids have circulated worldwide. Sun et al. (2017) also reported successful transport of the *mcr-1* gene by IncX4 plasmids (9/11) in Enterobacteriaceae. The second most frequently identified plasmid was IncHI2 carrying the *mcr-1* gene in 21% (13/61) of *E. coli* isolated from turkey meat samples from Poland (*n* = 5). These plasmids were transferable in 8 out of 13 isolates tested. The IncI2-type plasmid carrying *mcr-1* was detected occasionally, and only in *E. coli* (3) and *C. braakii* (1) isolates from rabbit meat samples from China (*n* = 2). The conjugation with IncI2 plasmid was successful in all these isolates. The *mcr-1* gene in *E. coli* isolated from rabbit meat in Portugal was also localized on an IncI2-type plasmid (Freitas-Silva et al., 2018).

The majority of *E. coli* as well as *K. pneumoniae* and *C. braakii* isolates harboring the *mcr-1* gene carried genes encoding co-resistance to several classes of antimicrobial agents (Supplementary Table 1). The high level of resistance to beta-lactams (88%) and tetracycline (85%) in Enterobacteriaceae with *mcr-1* from meat that was found in this study is consistent with the results of antibiotic resistance in food-producing animals in other European countries (El Garch et al., 2018). In particular, the presence of plasmid-mediated CT resistance in multidrug-resistant bacteria, such as ESBL producers, poses a significant threat to public health. Indeed, in our study, ESBL-encoding genes were detected in 18% (11/61) of isolates carrying the *mcr-1* gene and belonging to *E. coli*, *K. pneumoniae*, and *C. braakii* isolated from meat from Poland, Germany, Czechia, Brazil, and China. The occurrence of plasmid-mediated CT resistance in ESBL producers isolated from meat is also described in other studies. In China, the *mcr-1* gene was found in 27% of ESBL-producing *E. coli* isolated from retailed meat and shrimps (Liu et al., 2017). In Denmark, the *mcr-1* gene was detected in 1.5% of ESBL-producing *E. coli* isolated from imported retailed chicken meat without declaration of origin (Kluytmans-van den Bergh et al., 2016). In a Brazilian study, most *E. coli* isolates (7/8) from chicken meat and liver were shown to carry some of the ESBL-encoding genes (Monte et al., 2017).

In our study, the fosfomycin resistance gene *fosA3* and genes for the production of ESBL (*bla*_CTX–M–__14_) and PMQR (*oqxA*, *oqxB*) were also detected in one *E. coli* isolate carrying the *mcr-1* gene, which was obtained from rabbit meat from China. The use of fosfomycin is advised for the treatment of both urinary and systemic infections caused by multidrug-resistant *E. coli*. The chromosomal *fosA* gene is often detected in *K. pneumoniae* (99.7%) but only occasionally in *E. coli* (4.6%). The plasmid-mediated *fosA3* gene variant is the most commonly reported in *E. coli* and other Enterobacteriaceae in Asia (Ito et al., 2017). Liu et al. (2017) detected the *fosA3* gene in seven ESBL-producing *E. coli* strain carrying the *mcr-1* gene that were isolated from food of animal origin. The occurrence of fosfomycin resistance in Asia, but also its sporadic detection in strain of animal origin in Europe (Lupo et al., 2018), represents another public health concern.

## Conclusion

The results of this study confirm the impact of global trade in raw meat on the spread of plasmid-mediated CT resistance. Turkey meat and liver were the most common sources of Enterobacteriaceae carrying the *mcr-1* gene when compared to other meats (other poultry, pork, beef, and rabbit). Despite the high heterogeneity of the isolates tested, the *mcr-1* gene was primarily localized on an IncX4-type plasmid, regardless of ST or the country of meat origin. The co-resistance of plasmid-mediated CT resistance encoded by the *mcr-1* and ESBL genes was 18%. Another noteworthy finding was the *fosA3* gene coding for fosfomycin resistance in a multidrug-resistant isolate of *E. coli* from rabbit meat imported from China. These results point to the threat posed by the dissemination of multidrug-resistant bacteria through the food chain. The increased prevalence of *E. coli* and *K. pneumoniae* isolates with the *mcr-1* gene in turkey meat support the need for Europe-wide monitoring of plasmid-mediated CT resistance in farm animals, especially in poultry, and in slaughterhouses.

## Data Availability Statement

The datasets generated for this study can be found in the European Nucleotide Archive (ENA) at 585 EMBL-EBI under accession number PRJEB34874 586 (https://www.ebi.ac.uk/ena/data/view/PRJB34874).

## Author Contributions

TG and RK performed the experimental planning and prepared the manuscript. LP and IK collected the isolates and carried out the experiments. AB and MF analyzed the whole-genome sequencing data. IJ performed the whole-genome sequencing, and MZ performed the conjugation assays in selected isolates. All authors discussed the results.

## Conflict of Interest

The authors declare that the research was conducted in the absence of any commercial or financial relationships that could be construed as a potential conflict of interest.

## Footnotes

1 https://cge.cbs.dtu.dk/services/SPAdes/

2 http://enterobase.warwick.ac.uk/species/index/ecoli

3 https://bigsdb.pasteur.fr/klebsiella/klebsiella.html

4 https://cge.cbs.dtu.dk/services/

5 https://enterobase.warwick.ac.uk/species/ecoli/download_data

6 https://www.cgmlst.org/ncs/schema/2187931/
